# Homocysteine promotes ferroptosis through NCOA4-mediated ferritinophagy in THP-1 macrophages

**DOI:** 10.3389/fphys.2026.1905092

**Published:** 2026-08-12

**Authors:** Yu Yang, Weiya Zhang, Dandan Huang

**Affiliations:** Institute of Basic Medicine, North Sichuan Medical College, Nanchong, Sichuan, China

**Keywords:** atherosclerosis, ferritinophagy, ferroptosis, homocysteine, THP-1 cells

## Abstract

**Introduction:**

As an independent risk factor for atherosclerosis (AS), hyperhomocysteinemia (HHcy) exerts its pathogenic effects primarily through the induction of macrophage ferroptosis. As a selective autophagic process mediated by nuclear receptor coactivator 4 (NCOA4), ferritinophagy directly influences ferroptosis via its regulation of cellular iron balance. However, whether homocysteine (Hcy) regulates macrophage ferroptosis through ferritinophagy remains unclear.

**Methods:**

Human acute monocytic leukemia (THP-1) cells were differentiated into macrophages and subsequently treated with Hcy. Ferroptosis was assessed by measuring glutathione (GSH) levels, reactive oxygen species (ROS), Fe²⁺ content, and mitochondrial morphology. Protein expression of NCOA4, FTH1, and GPX4 was examined, and GPX4 methylation was evaluated. The involvement of ferritinophagy was further verified using the ferroptosis inhibitor ferrostatin‑1 (Fer‑1) and the inducer Erastin. Activation of the IL‑6/STAT3 signaling pathway was also examined, along with its reciprocal regulation with ferroptosis.

**Results:**

Hcy treatment promoted ferroptosis in THP‑1 macrophages, as indicated by decreased GSH levels, increased ROS and Fe²⁺ levels, and characteristic mitochondrial morphological changes. Hcy also enhanced GPX4 methylation, resulting in reduced GPX4 expression. Mechanistically, Hcy upregulated NCOA4 and downregulated FTH1, suggesting activation of NCOA4‑mediated ferritinophagy; these effects were reversed by Fer‑1 and augmented by Erastin. In addition, Hcy activated the IL‑6/STAT3 pathway, and its crosstalk with ferroptosis was confirmed by the reciprocal modulation with Fer‑1 and Erastin.

**Discussion:**

Collectively, our study indicates that Hcy promotes ferroptosis in THP-1 macrophages through NCOA4-mediated ferritinophagy, and the IL-6/STAT3 signaling pathway plays a key role in this process. Therefore, targeting Hcy may represent a potential treatment strategy for AS.

## 1 Introduction

Atherosclerosis (AS) constitutes a major risk factor for a range of cardiovascular diseases, with its hallmark features including lipid deposition, monocyte infiltration, and inflammatory responses (Libby, 2021). The pathophysiology of atherosclerotic disease is complex and progressive (Blaha et al., 2023). It mainly results from endothelial cell dysfunction and the lipid accumulation leading to plaque formation. During this process, vascular smooth muscle cells and macrophages are the focus of attention (Liu et al., 2025). Interestingly, an increasing number of studies have pointed out macrophages play an essential position in the AS, and its development is deeply influenced by their inherent plasticity (Feng et al., 2025).

As a sulfur-bearing amino acid, homocysteine (Hcy) functions as a metabolic intermediate in the methionine catabolic pathway (Agostini et al., 2025). Elevated blood levels of Hcy indicate hyperhomocysteinemia (HHcy) and have been regarded as a dangerous factor for various diseases (Li et al., 2024). HHcy promotes oxidative stress, the superfluous accumulation of reactive oxygen species (ROS), and contributes to mitochondrial dysfunction and endothelial cell injure, thus making it a key pathogenic factor for cardiovascular diseases (Kaplan et al., 2020; Yuan et al., 2023). Additionally, through disruption of the SAM/SAH dynamic balance, HHcy exerts an epigenetic effect that leads to DNA methylation, thereby influencing the progression of AS disease (Dai et al., 2022). Many studies show that HHcy is an unequivocally recognized significant independent pathogenic factor for AS (Tian et al., 2025). Nevertheless, the precise molecular mechanisms underlying the influence of Hcy in AS progression remain to be fully elucidated.

Ferroptosis is a form of regulated cell death characterized by abnormal iron metabolism and lipid peroxidation (Dixon et al., 2012), and its impact on AS is mediated by various signaling pathways (Vinchi et al., 2019; Ouyang et al., 2021). Within AS lesions, macrophages serve as central mediators of inflammatory responses, plaque development, and thrombotic events (Xue et al., 2023)while also playing an essential role in iron homeostasis. Additionally, macrophage ferroptosis is recognized as a critical component of cell death within atherosclerotic plaques (Li et al., 2022; Ru et al., 2024). In light of these observations, investigating the association between macrophage ferroptosis and AS could provide new therapeutic avenues for AS.

Autophagy is an evolutionarily conserved catabolic process essential for maintaining cellular homeostasis and promoting cell survival under stress. Three main types of autophagy exist in cells: microautophagy, chaperone-mediated autophagy, and macroautophagy (Kundu et al., 2025; Wang et al., 2026). Ferritinophagy represents a selective form of macroautophagy, characterized by the NCOA4 (nuclear receptor coactivator 4)-mediated delivery of ferritin (FTH1/FTL complex) to the autolysosome for degradation, leading to the release of ferrous iron (Fe^2+^). As a cargo receptor, NCOA4 not only links the ferritin complex to the core autophagic machinery but also serves as a molecular nexus that integrates iron metabolism, autophagy, and regulated cell death (Chabane et al., 2026; Chen et al., 2026). NCOA4 binds to the ferritin heavy chain 1 (FTH1) subunit, leading to the release of stored iron and thereby modulating ferroptosis through iron homeostasis (Hoelzgen et al., 2024; Zhao et al., 2025; Zhong et al., 2025). Indeed, recent literature indicates that selective autophagy can be regulated separately from bulk autophagy (Liu et al., 2020). Meanwhile, emerging research has proved that ferritinophagy and ferroptosis occupy important status in the progress of cardiovascular disease (Qin et al., 2021). Our previous study demonstrated that Hcy reduces autophagic vesicle accumulation, upregulates p62 expression, and downregulates LC3 and Beclin-1 expression, thus indicating that Hcy exerts an inhibitory effect on autophagy in macrophages (Yang et al., 2022a). However, whether Hcy modulates ferritinophagy remains unclear. Therefore, targeting the effect of Hcy on ferritinophagy and ferroptosis establish a fresh idea for the therapy of AS.

Based on these preliminary findings and the existing literature, the current study was designed to investigate the role of ferroptosis in macrophages following Hcy treatment. Furthermore, the potential involvement of NCOA4-mediated ferritinophagy in atherogenesis was examined.

## 2 Materials and methods

### 2.1 Reagents and antibodies

GAPDH(ET1601), GPX4(ET1706), NCOA4(HA722282), FTH1(ET1610), p-STAT3(Y705), STAT3(ET1605), and IL-6(R1412-2) were purchased from HuaBio Tech (Shanghai, China). The secondary antibody anti-rabbit IgG was acquired from Proteintech ^®^ (Cat#SA00001-2, Wuhan, China). The primary assay kits and reagents used were PE anti-human CD11b antibody (clone ICRF44, Cat# 301306) and FITC anti-human CD14 antibody (clone HCD14, Cat# 325604) from BioLegend (CA, USA), DL-homocysteine (H4628, Sigma, USA), Cell Counting Kits-8 assay (#CT0001-B, SparkJade, China), ferrostatin-1(Fer-1) (CSN12654, CSNpharm, Arlington Heights, IL, USA), Erastin (CSN17148, CSNpharm, Arlington Heights, IL, USA), and phor-bol-12myristate13acetate (PMA) (S1819-1mg, Beyotime, Shanghai, China). The total glutathione (T-GSH)/oxidized glutathione (GSSG) Colorimetric Assay Kit (E-BC-K097-M), Reactive Oxygen Species (ROS) Fluorometric Assay Kit (E-BC-K138-F), and Cell Ferrous Iron Colorimetric Assay Kit (E-BC-K881-M) were obtained from Elabscience Biotechnology Co., Ltd. (Wuhan, China); Animal Tissue/Cell Genome DNA Extraction Kit (D1700) from Solarbio Science & Technology; China. ChamQ Universal SYBR qPCR Master Mix (Q711-02) and HiScript^®^ III RT SuperMix for qPCR (+gDNA wiper) (R323-01) from Vazyme Biotech (Nanjing, China).

### 2.2 Cell culture and intervention

Human peripheral monocytic THP-1 cells were purchased from Procell Life Science Tech (Wuhan, China). The cells were cultured in RPMI-1640 medium (#L210KJ, Basal Media, China) supplemented with 1% penicillin–streptomycin (#C125C5, NCM Biotech, China), 10% FBS (#C04001, Viva Cell, China), and 0.05 mM β-mercaptoethanol (M8211, Solarbio Science & Technology, China). Subsequently, the cells were grown at 37 °C in an incubator containing 5% CO2. Following a 48h incubation with 100 ng/mL PMA, THP-1 derived-macrophages were successfully induced.

### 2.3 Staining for flow cytometry

THP-1 cells were seeded in 6-well plates at a density of 1 × 10^6^ cells/mL, with each well containing 2 mL of RPMI-1640 medium supplemented with 100 ng/mL PMA. After treatment for 48 h, the culture medium was removed, and THP-1 macrophages were washed with PBS twice. The cells were then harvested in a flow tube after trypsinization and centrifugation. We used CD14 FITC and CD11b PE as surface markers of THP-1-derived macrophages. According to the manufacturer’s instructions, after being incubated with an ultimate density of 10 μM CD14 FITC and CD11b PE at 4 °C away from light for 30 min, the pellet was suspended in PBS. Samples were performed using Cyto FLUX Ultimate flow cytometry (Legend Teck, China) and quantified via Flow Jo software.

### 2.4 Cell viability assay

The cells were counted and inoculated into each well of a 96-well culture plate at a density of 1×10^5^ cells/well After incubation with PMA for 48 h, they were administered with the corresponding concentrations of Hcy (25, 50, 100, 250, 500, 1000, 2000, and 5000 μM), with 3 replicate wells for every treatment group. Following 24 h of treatment, PBS was used to washed the cells twice. Each group was subsequently incubated with 100 μL of RPMI-1640 and 10 μL of CCK-8 working solution for 1 h at 37 °C. The absorbance at 450 nm was recorded on a microplate reader (Thermo, USA).

### 2.5 Transmission electron microscopy

The cells were digested with trypsin, rinsed twice with PBS, and then fixed overnight in 2.5% glutaraldehyde at 4°C. Subsequently, the cells were fixed with cacodylate-buffered 1% osmium tetroxide for 1.5 hours at 37°C. Thereafter, the fixed cells were dehydrated stepwise with a concentration grade of acetone: 30%, 50%, 70%, 80%, 90%, 95%, and 100% (fixed with a 100% concentration of acetone 3 times). Infiltration was performed using a desiccant and Epon-812 embedding agent in 3:1, 1:1, and 1:3 proportions. Following this, the Epon-812 pure embedding agent was used for polymerization. Ultrathin sectioning was then conducted (Leica UC7rt ultrathin microtome, Japan), which was followed by staining with uranyl acetate for 30 min and lead citrate for 1–2 minutes at room temperature. The mitochondrial structure was caught under a JEOL JEM-1400FLASH transmission electron microscope.

### 2.6 Detection of iron content

Intracellular ferrous iron (Fe^2+^) content was measured with a Cell Ferrous Iron Colorimetric Assay Kit according to the manufacturer’s instructions. In brief, the 96‑well plate was supplemented with 80 μL of both the standard and the test samples. Thereafter, the test samples were used to establish the control and test groups. Subsequently, 80 μL of control solution was added to the control groups, while the test and standard groups were treated with a chromogenic solution. The reaction mixtures were thoroughly mixed for 15 min in indoor temperature while being avoided light exposure. Then, absorbance was detected at 593 nm.

### 2.7 Reactive oxygen species assay

DCFH-DA (2,7-dichlorofluorescein diacetate) serves as a cell‑permeable fluorescent probe that itself exhibits no fluorescence. In the presence of ROS, DCFH is oxidized to DCF (dichlorofluorescein), a highly green‑fluorescent compound that is impermeable to cell membranes. THP-1 cells were seeded in 6-well plates at a density of 1 × 10^6^ cells/mL, with each well containing 2 mL of RPMI-1640 medium supplemented with 100 ng/mL PMA. Following a 48-hour induction, the cells were treated with graded concentrations of Hcy (25, 100, 250, and 500 µM) for 24 hours. Then, the cells were incubated with DCFH-DA (10 μM) at 37 °C for 30 min and protected from light, then immediately analyzed on a fluorescence microscope.

### 2.8 Determination of glutathione level

The total GHS level in macrophages was analyzed using a total glutathione (T-GSH)/oxidized glutathione (GSSG) Colorimetric Assay Kit, following the manufacturer’s instruction. After the cells were treated, 400 μL of reagent 3 was added to 1×10^6^ cells and ultrasonically crushed to collect cells. After centrifugation at 4 °C 10000×g for 10 min, the supernatant was extracted and mixed with 150 μL of the reaction working solution for 5 min and 50 μL of reagent 9 for 25 min. Detection and quantification of absorbance at 412 nm was performed.

### 2.9 Quantitative real-time PCR assay

TRIzol^®^ Reagent (#15596026, Thermo Fisher, USA) was used to isolate intracellular RNA, total RNA (1 μg) was reverse-transcribed to cDNA by using HiScript^®^ III RT SuperMix for qPCR (+gDNA wiper) kit, and ChamQ Universal SYBR qPCR Master Mix for obtaining cDNA by quantitative polymerase chain reaction (qPCR). The gene expression levels of β-actin, GPX4, NCOA4, FTH1, and STAT3 were assessed via qRT-PCR. Additionally, using β-actin as the control gene, the target mRNA transcript results were measured via the 2^ΔΔCt^ way. The specific primer information is as follows (**Table 1**).

**Table 1:** The primer information.

Gene name	F	R
β-actin	CATGTACGTTGCTATCCAGGC	CTCCTTAATGTCACGCACGAT
GPX4	CGCTGTGGAAGTGGATGAAGATC	TGTCGATGAGGAACTGTGGAGAG
FTH1	AGAACTACCACCAGGACTCAGAGG	AGCCACATCATCGCGGTCAAAG
NCOA4	TTGCATAAGCCGTCACCTGGAATG	GCCTGCTGTTGAAGTGTCTCCTC
STAT3	CTGTGGGAAGAATCACGCCT	ACATCCTGAAGGTGCTGCTC
GPX4-MSP(M)	AGTATTTTTAGGTTGTT TGGTTTGC	CGAACGTACGAACTTATTATTAACGA
GPX4-MSP(U)	GTATTTTTAGGTTGTTT GGTTTGTG	CAAACATACAAACTTATTATTAACAC

### 2.10 Methylation-specific PCR

The proteinase K method was used to isolate genomic DNA from THP-1 macrophages. Bisulfite conversion was conducted utilizing a DNA bisulfite conversion kit (EM102-01, Vazyme, China). After that, unmethylated cytosines were changed to uracils, whereas methylated cytosines were protected from this change. Based on the methylation preference sites of the GPX4 promoter, Sangon Biotech (Shanghai, China) designed and synthesized the primers. As shown in **Table 1**, MSP used two types of primers. One primer pair specifically targeted fully methylated sequences, recognizing and binding to the cytosines that were not converted during the bisulfite treatment process, while the other type was used for fully unmethylated sequences, binding to the uracils derived from the cytosines. According to the protocols of 2×EpiArt HS Taq Master mix (Dye Plus) (EM202-01, Vazyme, China), the MSPs contained 2×EpiArt HS Taq Master mix, two types of primers, bisulfite-converted DNA eluate, and ddH_2_O were combined for real-time PCR amplification. After amplification, electrophoresis on 1% agarose gel with Gel‑Red staining was employed to analyze the MSP products, followed by ultraviolet illumination for visualization.

### 2.11 Western blotting

The cell suspension at a concentration of 1×10^6^ cells/mL was seeded in a 6-well plate, followed by treatment with PMA for 48 h and then Hcy (25, 100, 250, and 500 µM) for 24 h. RIPA buffer fortified with protease inhibitors (cocktail) and 1mM PMSF (Beyotime, China) was employed to isolate total proteins from macrophages. The protein concentration of cells was detected by BCA protein quantitative reagent (E112, Vazyme, China). Cell lysates were distributed using SDS-PAGE and moved to PVDF membranes (Millipore, China). Subsequently, the PVDF membranes were combined with 5% protein blocking solution at indoor temperature for 1 h, and the primary antibodies GAPDH (1:2000) GPX4(1:1000), FTH1(1:1000), NCOA4(1:1000), IL-6(1:1000), p-STAT3(1:1000), and STAT3(1:1000) were treated at 4 °C overnight. Three TBST rinses were performed, and then the secondary antibody (HRP-conjugated, 1:2000) was applied for 1 hour. Then, the ECL working solution was performed to visualize protein bands on Image Lab™ Soft-ware (Bio-Rad, USA), which were then quantified via ImageJ software. Each protein band quantification was repeated at least three times.

### 2.12 Statistical analysis

All data acquired based on more than three independent experiments were used GraphPad Prism (version 8.0) and analyzed with mean ± standard deviation (SD). Differences between two groups were assessed using student’s t‑tests, whereas one‑way analysis of variance (ANOVA) was applied for comparisons involving three or more groups. Statistical significance was established at P<0.05.

## 3 Results

### 3.1 THP-1 monocytes were induced into macrophages

**Figure 1:**
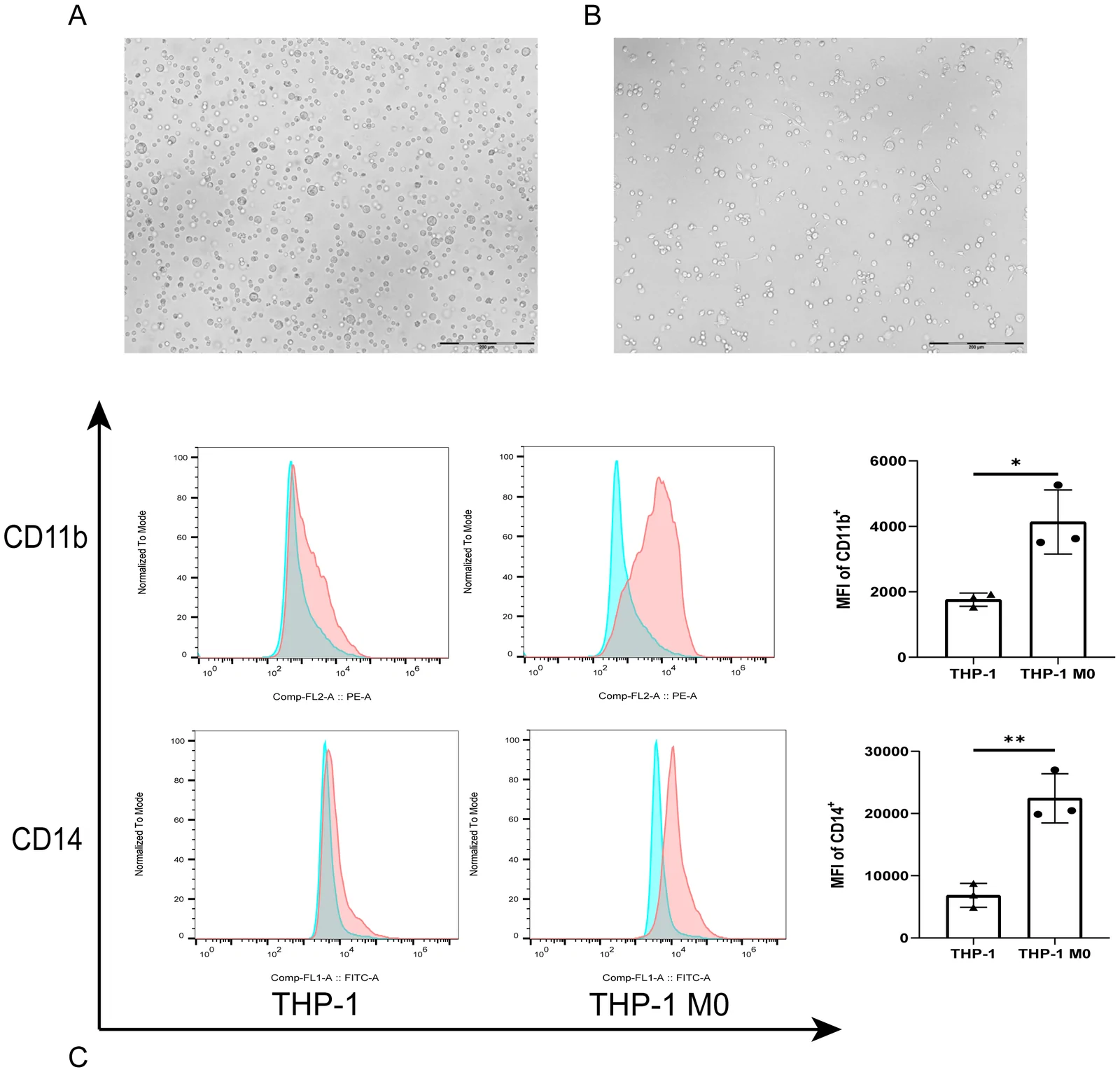
PMA induces the differentiation of THP-1 monocyte into macrophages. **(A)** THP-1 monocytes cultured with medium. **(B)** After 48 h of culture with PMA (100 ng/mL) in THP-1 monocytes. **(C)** The MFI (mean fluorescence intensity) of CD11b and CD14 was detected by Flow cytometry in THP-1 and THP-1 M0 (100 ng/mL PMA). *p<0.05; **p < 0.01 as opposed to THP-1 group.

We first used an inverted microscope to observe the morphological changes in cells. THP-1 monocytes were round with clear edges and predominantly exhibited a suspended growth state. The cells were plump, transparent, and highly refractive, with homogeneous cytoplasm (**Figure 1A**). Following stimulation with PMA, most cells adhered to the culture dish and acquired an irregular, spindle-shaped morphology with extended pseudopodia, all of which are hallmark features of macrophages (**Figure 1B**). Subsequently, flow cytometry analysis revealed the increased mean fluorescence intensity of CD11b and CD14 (**Figure 1C**), thus confirming PMA effectively induces THP-1 monocyte differentiation into macrophages.

### 3.2 Hcy enhances oxidative stress and promotes ferroptosis in macrophages

**Figure 2:**
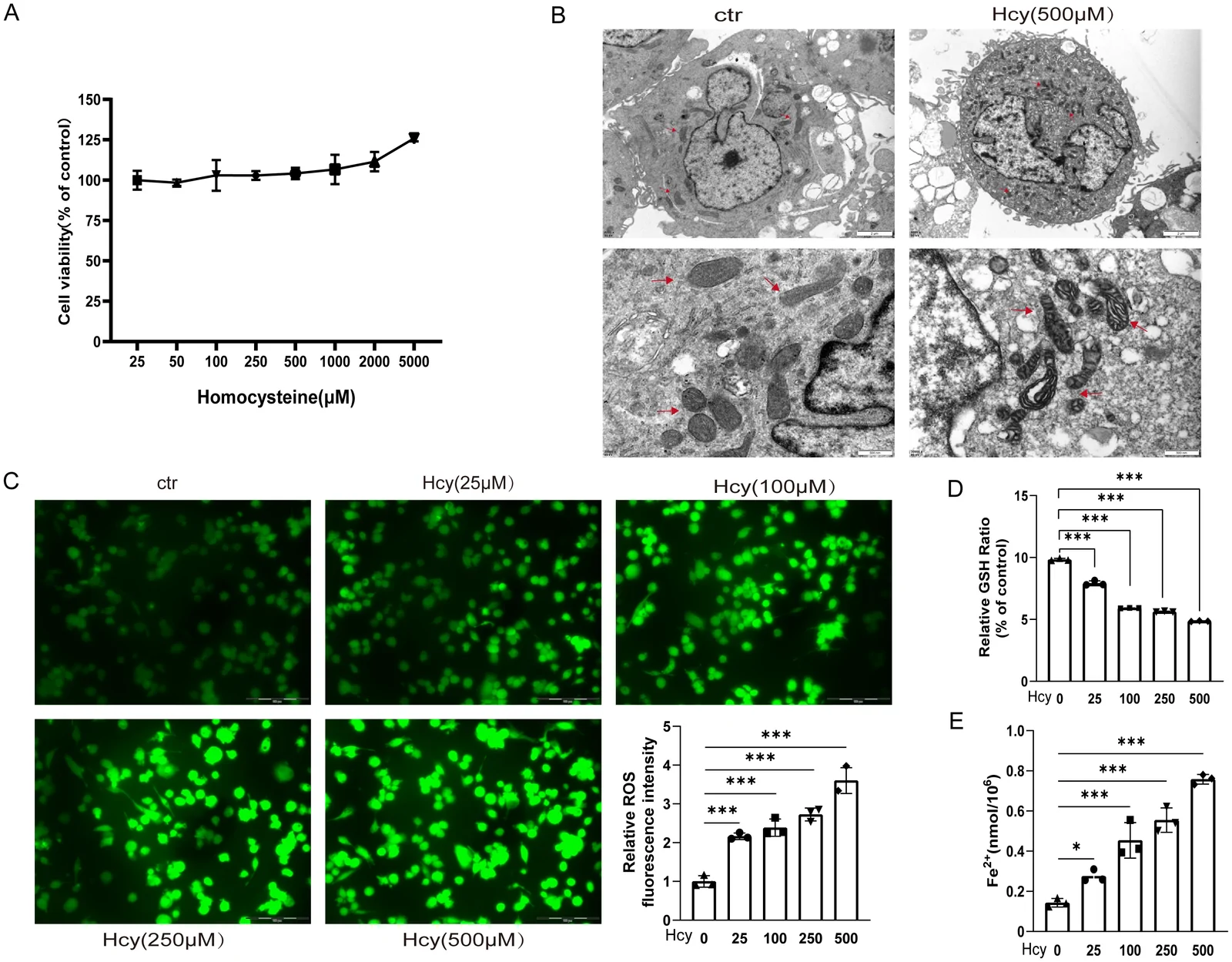
Cell viability and ferroptosis Following Hcy treatment. **(A)** Cell viability of THP-1 macrophages was quantified using the CCK-8 method. **(B)** Comparison of mitochondrial ultrastructure between control and Hcy (500 μM) groups via representative transmission electron micrographs. **(C)** The ROS level changes following different Hcy concentrations were detected using DCFH-DA fluorescence microscopy. **(D)** Changes in T-GSH levels with different concentrations of Hcy. **(E)** Intracellular ferrous iron content following different Hcy concentrations. *p<0.05; ***p < 0.00.1 as opposed to 0 group.

We evaluated the cytotoxicity of various concentrations of Hcy on macrophages over a 24 h period. The results indicated that none of the tested Hcy concentrations exhibited cytotoxic effects on macrophages within 24 h (**Figure 2A**). Plasma Hcy levels exceeding 15 μmol/L are defined as HHcy (Li et al., 2024), it is further categorized based on blood Hcy levels into mild (15–30 μmol/L), intermediate (30–100 μmol/L), and severe (>100 μmol/L) types (Marroncini et al., 2024). Congenital defects in Hcy metabolism can lead to markedly elevated blood Hcy levels, ranging from 200 to 300 μmol/L (Liu et al., 2017). Consequently, Hcy concentrations of 25, 100, 250, and 500 μM were selected for the subsequent experiments, with 500 μM serving as the primary experimental concentration.

**Figure 3:**
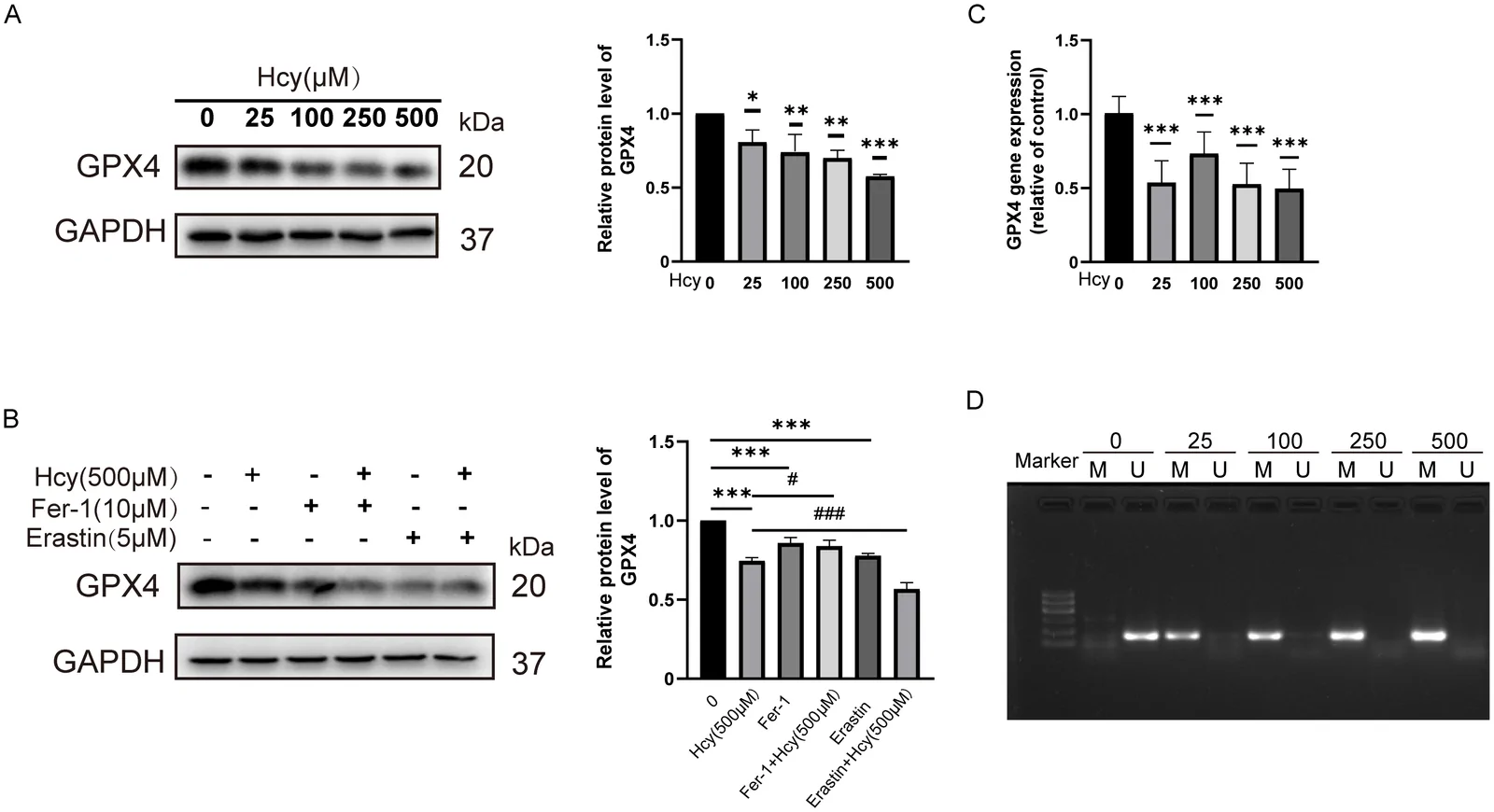
Impact of Hcy on GPX4 expression in THP-1 macrophages. **(A)** Western blotting bands of GPX4. **(B)** Protein expression levels of GPX4 after treatment with ferrostatin-1 and Erastin were analyzed via Western blotting. **(C)** GPX4 mRNA level changes. **(D)** GPX4 methylation alteration. M and U represent methylated and unmethylated GPX4 of Hcy-treated cells (25 μM, 100 μM, 250 μM, and 500 μM). *p<0.05; **p < 0.01; ***p< 0.001 as opposed to 0 group. *^#^p < 0.05*; *^##^p < 0.001* as opposed to Hcy group.

To investigate whether Hcy induces ferroptosis in THP-1 macrophages, we assessed mitochondrial damage using TEM and evaluated oxidative stress by measuring ROS, Fe^2+^, and GSH. TEM provides critical morphological evidence for identifying ferroptosis. As shown in the electron micrographs, compared with the control cells, mitochondria in Hcy-treated cells showed decreased volume, enhanced membrane density, and cristae rupture or absence—morphological changes characteristic of ferroptosis (**Figure 2B**). Meanwhile, ROS analysis indicated that Hcy significantly increased fluorescence intensity (**Figure 2C**). As the concentration of Hcy increased, intracellular Fe^2+^ levels progressively rose (**Figure 2E**). In contrast, GSH levels were found to decrease with increasing concentrations of Hcy in the treatment (**Figure 2D**). Further assessment was conducted on the expression of pivotal proteins involved in ferroptosis—GPX4, which is an essential glutathione-dependent regulatory factor in the ferroptosis regulatory pathway and serves as a pivotal downstream member in the inhibition of ferroptosis. Following Hcy treatment, the protein level of GPX4 was significantly reduced (**Figure 3A**). To delineate the function of ferroptosis in this process, we designed groups with the Erastin (5 μM) and Fer-1 (10 μM). Our results indicated that Fer-1 reversed the Hcy-induced downregulation of GPX4, whereas Erastin further potentiated this effect (**Figure 3B**). Consistent with the protein expression results, changes in GPX4 mRNA expression followed a similar pattern (**Figure 3C**). We further utilized a methylation-specific PCR (MSP) to detect the methylation status of the GPX4 gene. The results showed that increasing Hcy concentrations were associated with a progressive rise in the GPX4 methylation level in THP-1 macrophages, thus significantly enhancing the brightness of the methylated agarose gel band (M) (**Figure 3D**). These results indicate that Hcy can induce ferroptosis in THP-1 macrophages through GPX4 methylation.

### 3.3 Hcy promotes ferroptosis through NCOA4-mediated ferritinophagy

**Figure 4:**
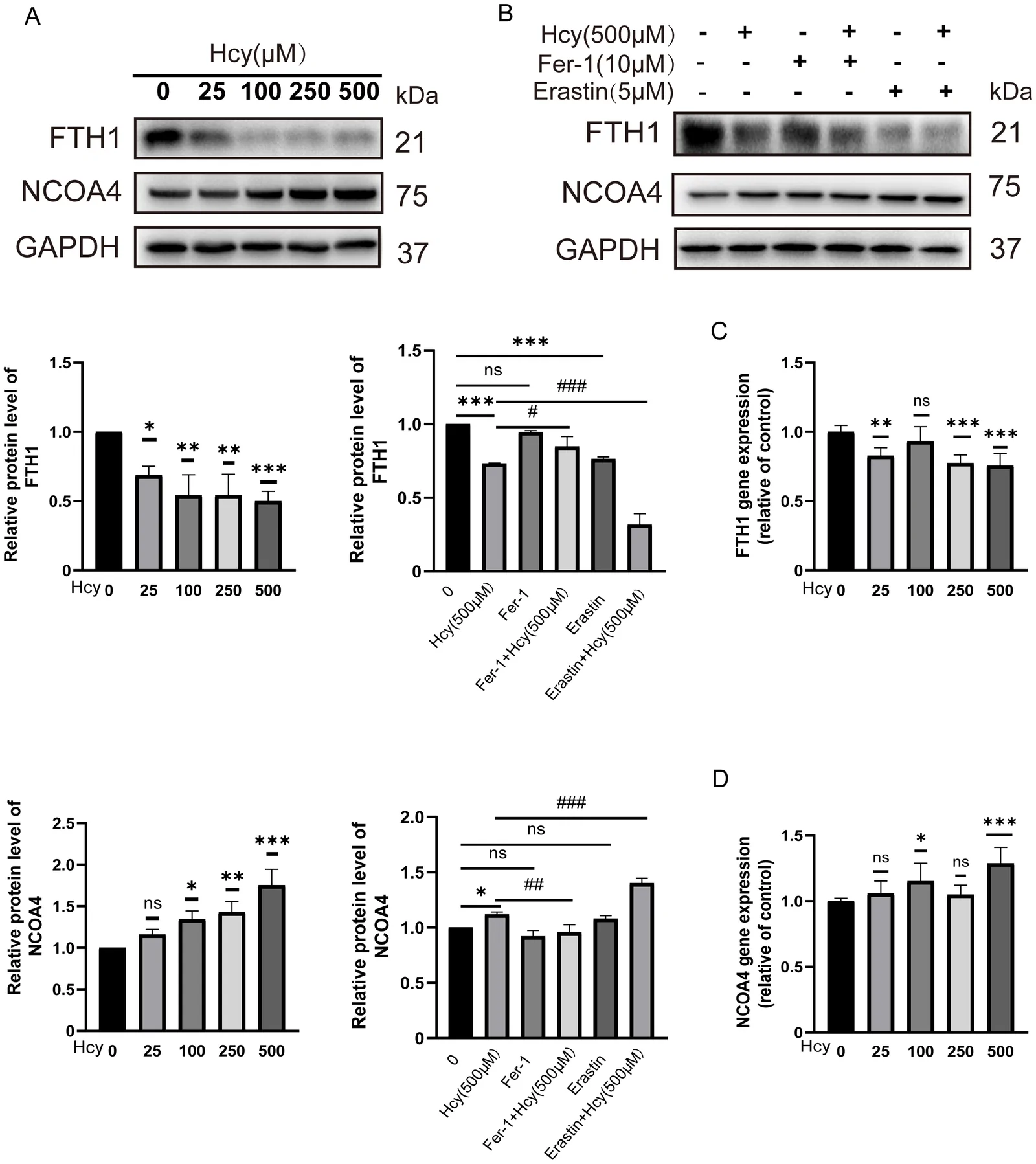
Impact of Hcy on FTH1 and NCOA4 expression in THP-1 macrophages **(A)** Western blotting bands of FTH1 and NCOA4. **(B)** The effects of ferrostatin-1 and Erastin on protein levels of FTH1 and NCOA4. **(C, D)** mRNA levels of FTH1 and NCOA4. *p<0.05; **p < 0.01; ***p < 0.001 as opposed to 0 group. *^#^p < 0.05*; *^##^p < 0.01*; *^###^p < 0.001* as opposed to Hcy group.

Previous studies have demonstrated that ferritin degradation and the consequent modulation of intracellular iron homeostasis are mediated by autophagy, which triggers the Fenton reaction and ultimately ferroptosis. This autophagy-dependent ferritin degradation, termed ferritinophagy, is regulated by NCOA4. Mechanistically, FTH1 directly interacts with NCOA4, and the resulting complex is targeted to autolysosomes for degradation. Uniformly, our results showed a dose-dependent reduction in FTH1 expression following Hcy treatment compared to the control group. Inversely, NCOA4 was obviously upregulated (**Figure 4A**). In contrast, NCOA4 was significantly upregulated. Subsequently, Fer-1 and Erastin served as pharmacological tools to further confirm the effect of Hcy in ferroptosis associated with NCOA4-mediated ferritinophagy. We found that the downward trend of FTH1 was reversed by Fer-1, whereas Erastin further enhanced it. In contrast, NCOA4 expression was significantly downregulated upon Fer-1 treatment, whereas it was markedly upregulated following Erastin treatment (**Figure 4B**). Consistently, qRT-PCR analysis demonstrated that Hcy increased NCOA4 gene expression in a dose-dependent manner, whereas FTH1 expression exhibited an inverse trend (**Figures 4C, D**).

### 3.4 Hcy promotes ferroptosis through the IL-6/STAT3 signaling pathway

**Figure 5:**
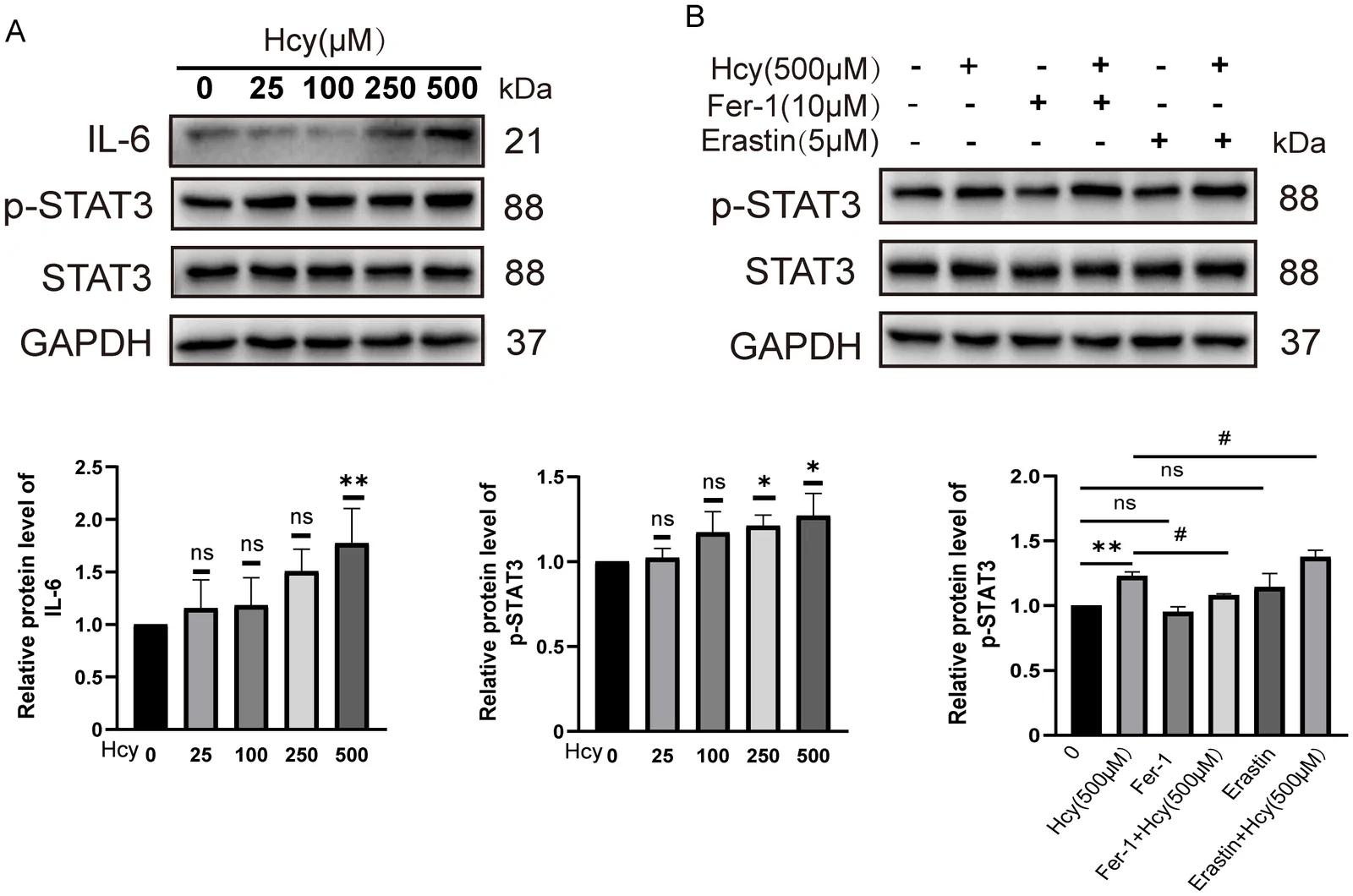
Effect of Hcy on expression of IL-6/STAT3 pathway-related proteins in THP-1 macrophages. **(A)** Western blotting bands of STAT3, p-STAT3 (Y705) and IL-6. **(B)** Protein expression of STAT3 and p-STAT3 (Y705) after treatment with ferrostatin-1 and Erastin. **(C)** Protein expression levels of p-STAT3 (Y705) after treatment with ferrostatin-1 and Erastin were analyzed using Western blotting. *p<0.05; **p < 0.05 as opposed to 0 group. *^#^p < 0.01* as opposed to Hcy group.

Atherosclerosis is a chronic inflammatory disease, and the IL-6/STAT3 pathway plays an important role in inflammation (Luo et al., 2021). Positive regulation of NCOA4 and augmentation of NCOA4- mediated ferritinophagy by STAT3 phosphorylation ultimately leads to lipid peroxidation and ferroptosis. Therefore, we sought to investigate whether Hcy regulates ferroptosis via the IL-6/STAT3 pathway. Imme (Zhu et al., 2023), we analyzed the protein expression levels of IL-6 and STAT3, and compared with control cells, Hcy treatment observably increased the protein expressions of IL-6 and p-STAT3, whereas total STAT3 protein level stayed unchanged (**Figure 5A**). The observations imply that the influence of Hcy on NCOA4-mediated ferroptosis is associated with the activation of the IL-6/STAT3 signaling pathway. Subsequently, we used Fer-1 and Erastin to further eliminate potential confounding factors. Consistent with our expectations, relative to the Hcy treatment group, the protein level of phosphorylated STAT3 was increased after Erastin treatment, whereas it was decreased after Fer-1 treatment. In contrast, total STAT3 expression remained unaffected (**Figure 5B**). In summary, all of the above data demonstrate that the IL-6/STAT3 signaling pathway may participate in NCOA4-mediated ferroptosis via Hcy and play a positive regulatory role.

## 4 Discussion

Our study confirms that Hcy promotes ferroptosis in human THP-1 macrophages, which is a process related to the IL-6-STAT3 signaling pathway, thereby enhancing our understanding of Hcy as a potential pathogenic factor in AS and the underlying mechanisms involved.

Ferroptosis is an iron overload-driven regulated cell death, and accumulating evidence indicates that ferroptosis is closely associated with the occurrence and progression of various diseases (Wu et al., 2021). Using single-cell sequencing, substantial positive of ferroptosis-related genes was detected in macrophages isolated from atherosclerotic plaques (Li et al., 2023). Recent studies have identified iron overload in macrophages, lipid peroxidation, intraplaque hemorrhage, and ROS accumulation as key features of advanced atherosclerotic plaques (Vinchi, 2021). These observations strongly suggest a close relationship between macrophages and ferroptosis, and raise the possibility that macrophage iron overload and ferroptosis play a role in AS progression (Ma et al., 2022; Yang et al., 2022b).

The atherogenic effects of Hcy are independent of other well-established atherosclerosis risk factors (Piazzolla et al., 2019). Moreover, multiple signaling pathways have been implicated in Hcy-mediated regulation of ferroptosis. Yang et al. (2025) found that Hcy induces endometrial ferroptosis via the MAPK pathway in recurrent pregnancy loss. Yin et al. (2025) reported that tectorigenin alleviates Hcy-induced inflammation and ferroptosis in BV-2 microglial cells via activation of the SIRT1/SLC7A11 pathway. Interestingly, Lei et al. (2025) demonstrated that Hcy promotes ferroptosis in cardiomyocytes in connection with the β-catenin/GPX4 pathway, thus suggesting that Hcy-induced ferroptosis may be closely related to AS. Consistent with this, we found that the Hcy treatment group exhibited a darker staining of mitochondrial chromatin and disrupted the mitochondrial crista structure, which are characteristic morphological features of ferroptosis. GSH is an abundant and stable antioxidant that donates electrons and scavenges various ROS (Alves et al., 2025). Conversely, excess iron can activate ROS production, thereby inducing lipid peroxidation in macrophages (Zhang et al., 2024). Our results showed that with increasing concentrations of Hcy, ROS levels and Fe^2+^ content gradually increased in THP-1 macrophages, whereas total GSH content progressively decreased. Consistent with the TEM data, these findings indicate that Hcy potentially facilitates macrophage ferroptosis through oxidative stress and mitochondrial damage.

As a critical modulator of ferroptosis, GPX4 contributes substantially to the maintenance of redox balance and contributing to the pathophysiology of several diseases, including ischemia–reperfusion injury and cardiovascular diseases (Liu et al., 2023; Zhang et al., 2024). Therefore, we measured GPX4 expression in Hcy-treated THP-1 cells. As expected, Hcy significantly reduced the expression of GPX4. To further validate the involvement of ferroptosis, we assessed GPX4 protein expression in THP-1 macrophages following treatment with Fer-1 or Erastin. Fer-1 inhibits ferroptosis by scavenging lipid peroxyl radicals and suppressing lipid peroxidation (Ji et al., 2024). Erastin exerts its ferroptosis‑inducing effect by inhibiting the cystine/glutamate antiporter system Xc^–^ (Li et al., 2020). Our results showed Fer-1 treatment significantly increased the expression of GPX4, whereas Erastin treatment decreased it. Research suggests that Hcy treatment inhibits GPX4 expression through increasing the promoter DNA methylation level (Pei et al., 2022). Moreover, studies demonstrated that enhanced GPX4 methylation mediates Hcy-induced upregulation of oxidative stress and ferroptosis in nucleus pulposus cells (Zhang et al., 2020). Our MSP results also indicated that Hcy enhances the methylation of GPX4 in THP-1 macrophages, and this effect shows a concentration-dependent increase. Therefore, it is plausible that Hcy modulates ferroptosis via inhibition of GPX4 methylation.

Emerging evidence indicates that ferritinophagy, a process in which NCOA4 carries ferritin to autophagolysosomes for degradation, serves as a key mediator in the etiology of cardiovascular diseases by regulating iron homeostasis and ferroptosis (Zhu et al., 2024; Wang et al., 2025). Notably, Zhu et al. (2023) found that NCOA4 protein expression increased while FTH1 decreased following oleic acid/palmitic acid treatment, thereby indicating that NCOA4-mediated ferroptosis plays a role in H9C2 cells. Corroborating these findings, our data indicated that the protein level of NCOA4 gradually increased with rising concentrations of Hcy. Conversely, the expression level of FTH1 exhibited a gradual downward trend following Hcy treatment. Subsequently, we validated these findings at the transcriptional level. The results showed that NCOA4 gene level gradually increased with rising Hcy concentrations, whereas that of FTH1 progressively decreased, which was consistent with the protein expression results. For further investigation into the function of Hcy in ferroptosis occurring via ferritinophagy, we treated cells with Fer-1 and Erastin. The findings demonstrated that Fer-1 attenuated the Hcy-induced upregulation of NCOA4 protein expression. Conversely, Fer-1 reversed the Hcy-induced downregulation of FTH1 protein expression. Meanwhile, Erastin treatment enhanced the Hcy-induced upregulation of NCOA4 protein expression while also exacerbating the Hcy-induced downregulation of FTH1 protein expression. Collectively, these results raise the possibility that Hcy promotes ferritinophagy-mediated ferroptosis in THP-1 macrophages.

Although our previous research demonstrated that Hcy inhibits autophagy in THP-1 macrophages via the AMPK-mTOR-TFEB signaling pathway (Yang et al., 2022a). As a cellular self-degradation and clearance mechanism, autophagy exhibits a dualistic function: it can confer protection during the initial phases of cellular injury, yet potentially exacerbate damage in the later stages (Tang et al., 2025). As a Moderate autophagy delays the progression of AS, whereas either insufficient or excessive autophagy may accelerate it (Zhu et al., 2024). Furthermore, the NCOA4-FTH1 axis functions as a major regulatory pathway controlling ferritinophagy and the cellular propensity for ferroptosis (Luo et al., 2025). However, detecting changes solely in NCOA4 and FTH1 expression is insufficient to confirm ferritinophagic flux. Ferritinophagy regulation involves cross-talk among iron metabolism, autophagy, oxidative stress, and lipid metabolism. The activity of the NCOA4-ferritinophagy pathway is not constitutive but is exquisitely regulated by a complex network of signaling pathways and molecular factors (Chen et al., 2026). This means that our exploration in this field still requires more time and effort.

IL-6 is an inflammatory cytokine involved in innate and adaptive immunity, neuronal maintenance, and metabolism (Rose-John, 2021). The sustained activation of IL-6 promotes endothelial dysfunction and AS (Lindkvist et al., 2022; Preda et al., 2025). As the primary downstream effector of the IL-6 signaling pathway, STAT3 is recognized as a key regulator of ferroptosis (Zhen et al., 2023; Xie et al., 2025). Studies have demonstrated that the modulation of the IL-6/STAT3 pathway, which in turn regulates NCOA4-mediated ferritinophagy, affects ferroptosis in vascular smooth muscle cells (Wang and Tang, 2024). Our results demonstrate that Hcy upregulates IL-6 protein level in THP-1 macrophages via a concentration-dependent manner. Meanwhile, Hcy increases the protein level of phosphorylated p-STAT3 without affecting total STAT3 expression. To further validate the role of ferroptosis, we treated cells with Fer-1 and Erastin. The results showed that Fer-1 inhibited the Hcy-induced upregulation of p-STAT3 protein expression, whereas Erastin enhanced this effect. Notably, total STAT3 protein expression remained unchanged following Fer-1 or Erastin treatment. In short, these findings indicate that Hcy activates the IL-6/STAT3 pathway, which may be associated with NCOA4-mediated ferritinophagy.

Nevertheless, a number of limitations associated with this study merit acknowledgment. Although our results preliminarily verify that Hcy promotes ferroptosis via the IL-6/STAT3 signaling pathway, how the pathway precisely regulates ferroptosis at the molecular level remains to be fully determined. Key unresolved issues include the crosstalk between GPX4 methylation and ferritinophagy activation, and whether NCOA4-mediated ferritinophagy might directly regulate ferroptosis through IL-6/STAT3 pathway. We should perform further experiments to exclude the potential influence of other factors on this process. Certainly, our conclusions are based solely on experiments using THP-1-derived macrophages; validation in clinical patient samples and *in vivo* animal models is warranted to establish the translational relevance of our findings.

## 5 Conclusions

In brief, we demonstrated that NCOA4-mediated ferritinophagy promotes ferroptosis in THP-1 macrophages and this process is related by the IL-6/STAT3 pathway. Further related experiments and animal experiments are warranted to elucidate the precise mechanism via which Hcy affects ferroptosis. Overall, this study highlights the important position of Hcy in the occurrence and progression of macrophage ferroptosis. From the perspective of ferritinophagy-mediated ferroptosis, deciphering the pathogenesis of AS may open up new possibilities for its intervention and treatment.

## Data Availability

The original contributions presented in this study are included in the article. Further inquiries can be directed to the corresponding author.
