# Supplementary material for: Homocysteine promotes ferroptosis through NCOA4-mediated ferritinophagy in THP-1 macrophages
Source: Front Physiol. 2026 Aug 12;17:1905092. doi: 10.3389/fphys.2026.1905092 (PMC13506723; doi:10.3389/fphys.2026.1905092)
Supplement: Supplementary file 1 [file DataSheet1.pdf]

Original blot images of western blots

In our western blot experiments, the proteins were added to the sample wells of 12% SDS-PAGE gel for electrophoresis with 80V for 30minutes, and then turn to 120V when the samples had passed through the upper gel. The whole gel was transferred to the polyvinylidene difluoride membrane at 180mA and running time was 2 hours. After that, the membrane was cut into two parts at around 25 kDa, and blocked with 5% non-fat dried milk at room temperature for 1 h, the membrane was incubated with GPX4(or other) antibody and GAPDH antibody mixed primary antibody solution at 4°C overnight. Then the membrane was incubated with horseradish peroxidase (HRP)-conjugated secondary antibody for 1hour at room temperature. the ECL working solution was used to visualize the protein bands on Image Lab™ Soft-ware (Bio-Rad, USA). The original pictures of blots are presented below.

Fig 3

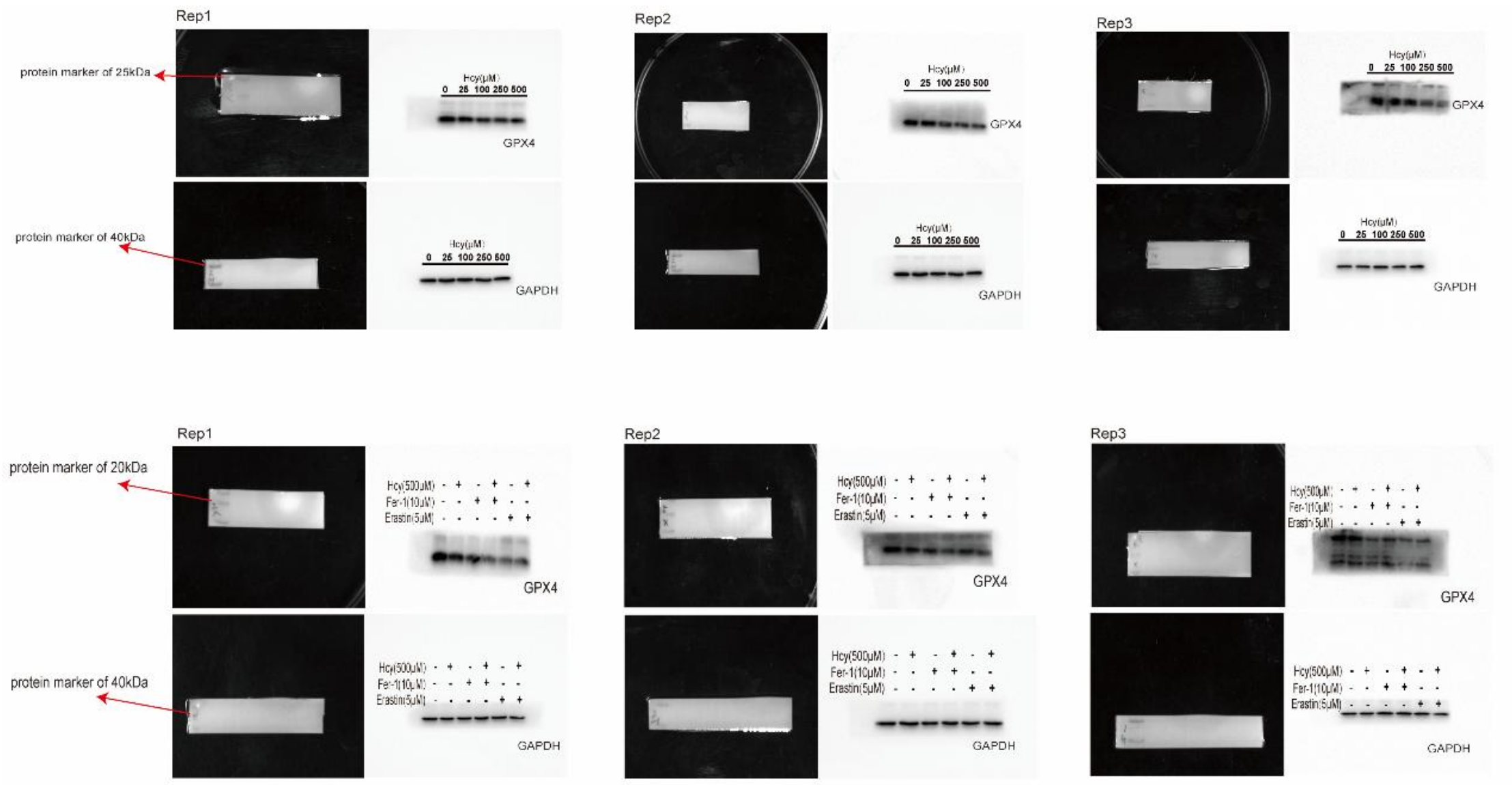

Fig 4

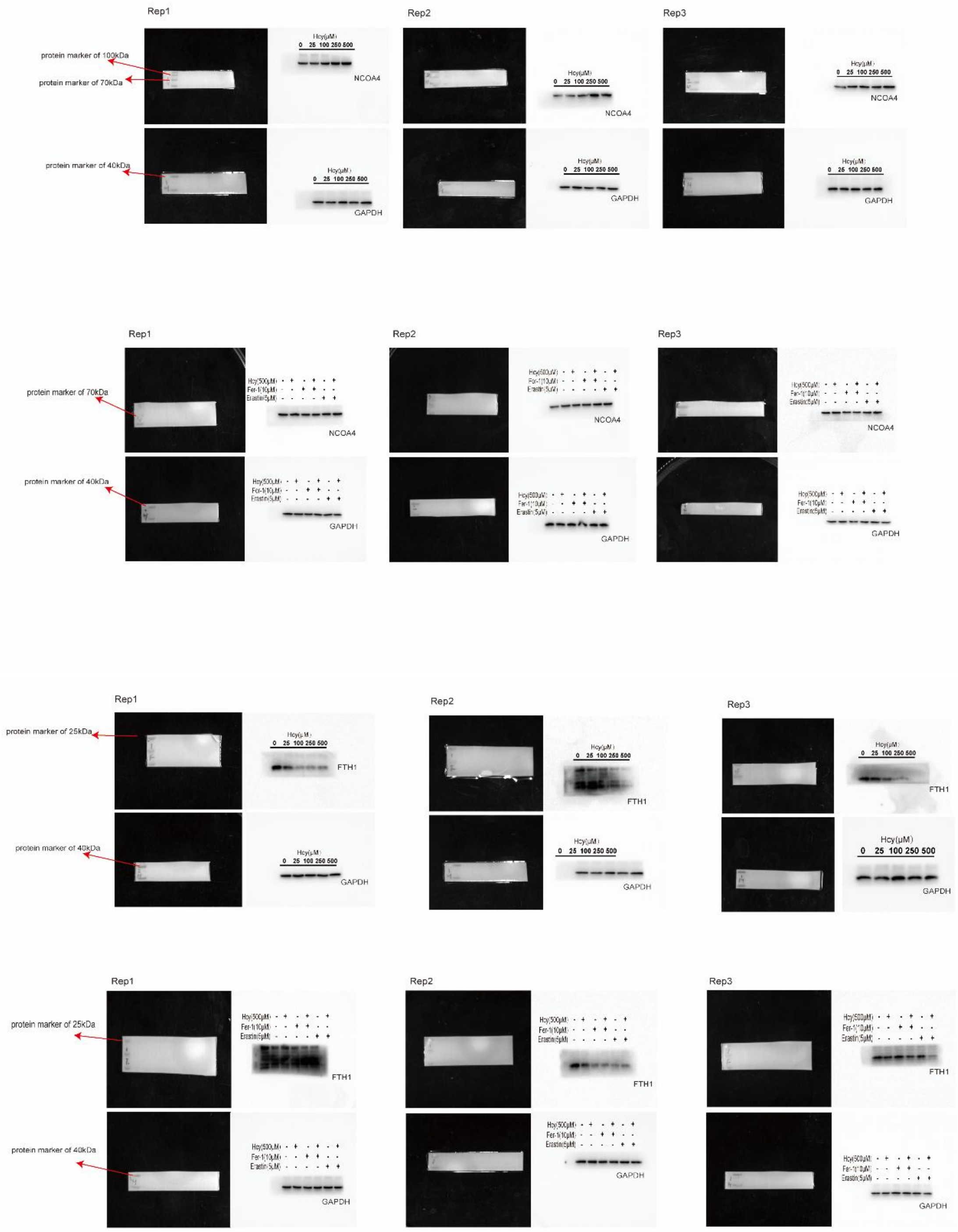
Rep1
Rep2
Rep3
protein marker of 100kDa
protein marker of 70kDa
protein marker of 40kDa
Hcy(μM)
0 25 100 250 500
NCOA4
GAPDH
Hcy(500μM)
Fer-1(10μM)
Erastin(5μM)
protein marker of 25kDa
FTH1

Fig 5

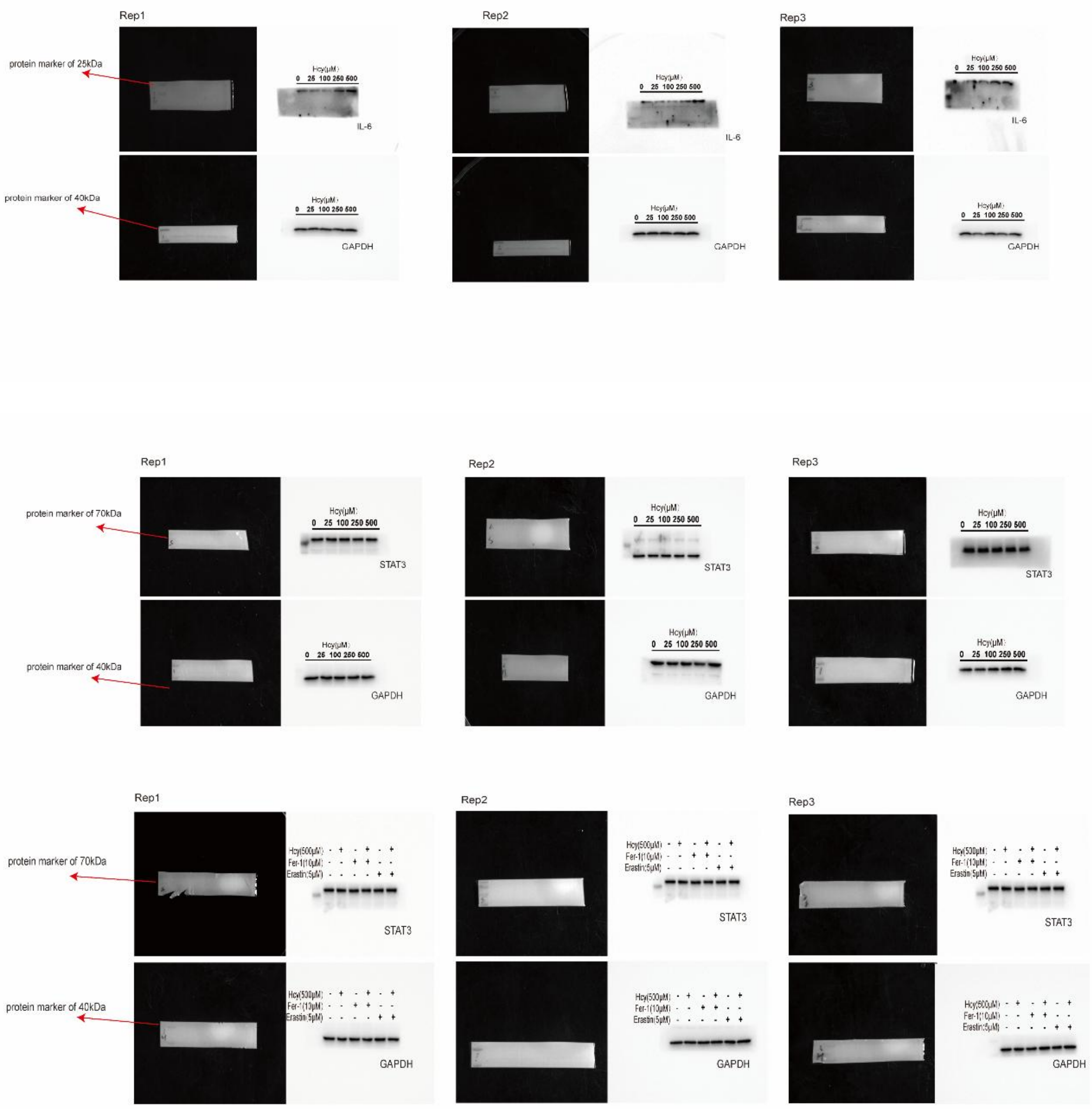
Rep1
Rep2
Rep3
protein marker of 25kDa
Hcy(μM)
0 25 100 250 500
IL-6
protein marker of 40kDa
GAPDH
Rep1
Rep2
Rep3
protein marker of 70kDa
Hcy(μM)
0 25 100 250 500
STAT3
protein marker of 40kDa
GAPDH
Rep1
Rep2
Rep3
protein marker of 70kDa
STAT3
protein marker of 40kDa
GAPDH

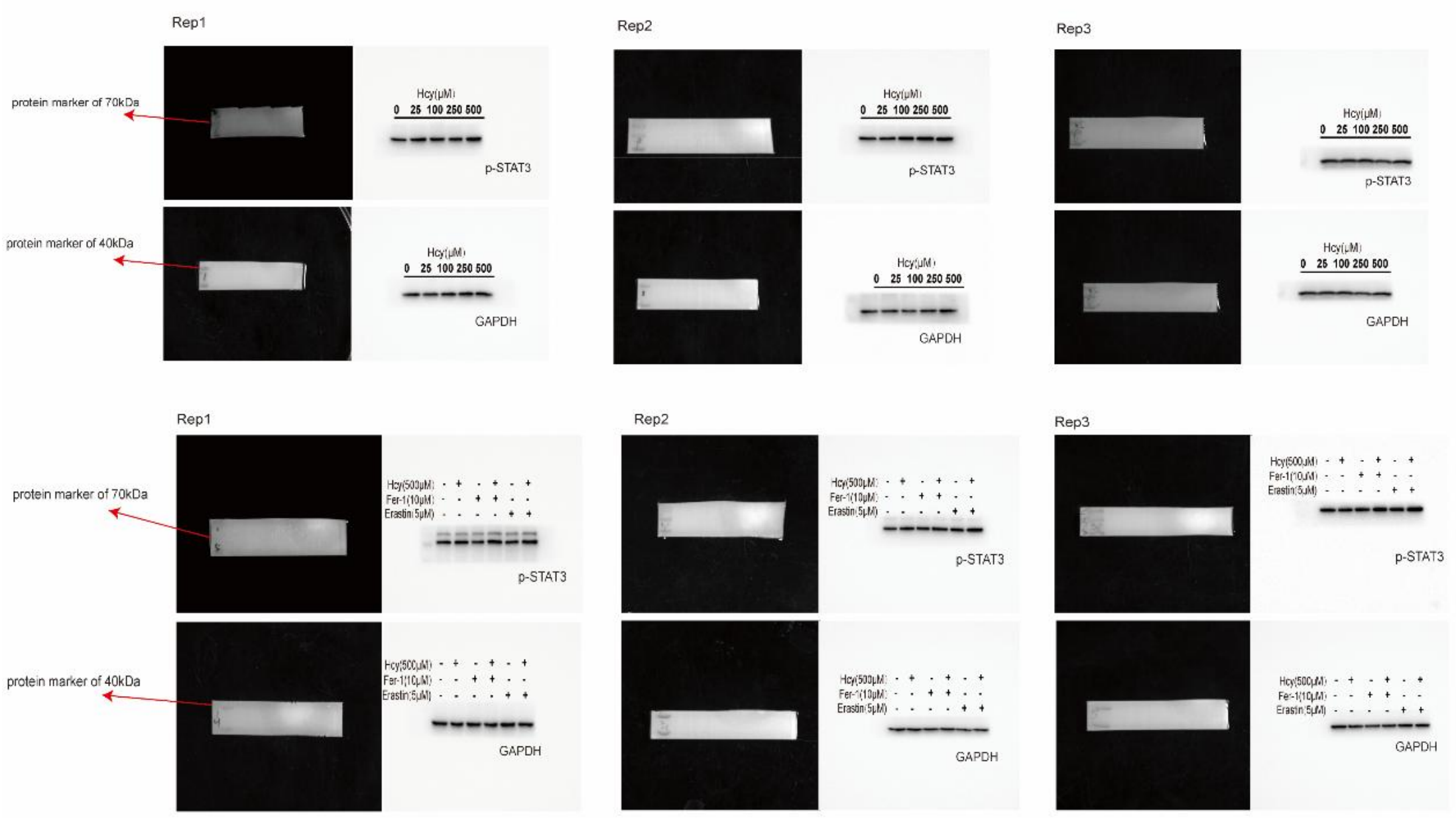

Rep1
Rep2
Rep3
protein marker of 70kDa
protein marker of 40kDa
Hcy(μM)
0 25 100 250 500
p-STAT3
GAPDH
Hcy(500μM)
Fer-1(10μM)
Erastin(5μM)
